# Real-World Outcomes of Nivolumab in Patients With Unresectable Hepatocellular Carcinoma in an Endemic Area of Hepatitis B Virus Infection

**DOI:** 10.3389/fonc.2020.01043

**Published:** 2020-06-30

**Authors:** Pil Soo Sung, Jeong Won Jang, Jaejun Lee, Soon Kyu Lee, Hae Lim Lee, Hyun Yang, Hee Chul Nam, Sung Won Lee, Si Hyun Bae, Jong Young Choi, Nam Ik Han, Seung Kew Yoon

**Affiliations:** ^1^The Catholic University Liver Research Center, College of Medicine, The Catholic University of Korea, Seoul, South Korea; ^2^Division of Gastroenterology and Hepatology, Department of Internal Medicine, College of Medicine, Seoul St. Mary's Hospital, The Catholic University of Korea, Seoul, South Korea; ^3^Division of Gastroenterology and Hepatology, Department of Internal Medicine, College of Medicine, Eunpyeong St. Mary's Hospital, The Catholic University of Korea, Seoul, South Korea; ^4^Division of Gastroenterology and Hepatology, Department of Internal Medicine, College of Medicine, Bucheon St. Mary's Hospital, The Catholic University of Korea, Seoul, South Korea

**Keywords:** hepatocellular carcinoma, nivolumab, objective response, tumor size, tumor heterogeneity

## Abstract

Real-world results of nivolumab monotherapy against HCC are lacking in the hepatitis B virus (HBV)-endemic, Asia-Pacific regions. Moreover, heterogeneous responses to immune checkpoint inhibitors have rarely been described in advanced HCC. The aim of this study is to evaluate the efficacy and safety of nivolumab monotherapy in a real-world setting in 33 Korean patients with unresectable HCC. In our cohort, twenty-nine patients (88%) showed HBsAg positivity. At the time of nivolumab initiation, 4 among 33 patients (12%) were classified as Barcelona Clinic Liver Cancer (BCLC)-B stage and 29 (88%) as BCLC-C stage, respectively. Prior sorafenib treatment was given to 31 (94%) patients, and 13 (39%) received prior regorafenib treatment. For the liver reserve, patients were classified as Child–Pugh class A (79%) and B (21%), respectively. Grade 3 toxicities occurred in one patient, who developed pneumonitis after 5 cycles of nivolumab treatment. Best overall responses were complete response in 2 patients out of the 33 enrolled patients (6%), partial response in 4 patients (12%) and stable disease in 4 patients (12%). With 29 patients having images for the response evaluation, the objective response rate was 21.4%. The median overall survival (OS) of the cohort was 26.4 weeks (range 2.3–175.1). Achieving objective responses, pre-treatment small tumors (maximal diameter <5 cm) and favorable liver function as assessed by Albumin–Bilirubin grade were significant factors for the favorable OS. Interestingly, differential responses to nivolumab among multiple tumors in a single patient were noted in 6 patients (18%). In these patients, small metastatic tumors were regressed, although their larger tumors did not respond to nivolumab monotherapy. In summary, nivolumab treatment seems clinically efficacious in treating unresectable HCC in an endemic area of HBV infection. Further prospective evaluation is required to overcome the heterogeneous efficacy of nivolumab monotherapy according to the baseline tumor burden.

## Introduction

Worldwide, hepatocellular carcinoma (HCC) is the fourth most common cause of cancer-related mortality. There are more than 850,000 new cases of liver cancer annually, 90% of which are HCC (1). Risk factors for HCC include chronic hepatitis B (CHB) and chronic hepatitis C (CHC), non-alcoholic fatty liver disease (NAFLD) and excessive alcohol ingestion (1). In Asian countries, where hepatitis B virus (HBV) infection is prevalent and accounts for 80% of victims, a considerable number of patients receive only supportive care or, at best, palliative treatments (2).

Sorafenib, a multi-tyrosine kinase inhibitor, has been the only drug available in the last decade to combat HCC (3). Recently, three tyrosine kinase inhibitors have demonstrated improved outcomes: lenvatinib in the first-line, and regorafenib and cabozantinib after first-line failure (4). Although they showed some promising results in terms of efficacy, their use may be limited due to the adverse effects and the potential decrease in the liver reserve. Immune checkpoint inhibitors are intended to target the programmed cell death protein-1 (PD-1), programmed death-ligand 1 (PD-L1), or cytotoxic T lymphocyte-associated protein-4 (CTLA-4) (5). Clinical trials with nivolumab and pembrolizumab in unresectable HCC, representative anti-PD-1 antibodies, had been anticipated to show prolonged survivals in patients treated with these drugs. However, only 14 to 18% of patients treated with pembrolizumab or nivolumab monotherapy had objective tumor responses (6–8). More recently, a phase 3 randomized, multi-centre study (CheckMate 459) evaluating nivolumab monotherapy versus sorafenib as a first-line treatment of unresectable HCC, did not achieve its primary endpoint of overall survival (OS) (9, 10). Moreover, unlike other solid tumors, there was no marked association identified between the levels of tumor cell PD-L1 expression and responses to nivolumab in HCC, reported by earlier Keynote-224 and CheckMate-040 studies (5, 11, 12). Currently, there are no validated biomarkers for HCC immunotherapy (13).

Recent sub-analysis of the CheckMate-040 study between intent-to-treat (ITT) overall population and an Asian cohort with prior sorafenib failure showed that treatment responses of Asian patients were similar to those of the overall population (14). This disappointing performance of nivolumab monotherapy may be attributed to the immune heterogeneity of HCC (15, 16). However, there is a lack of real-world clinical data demonstrating the heterogeneous responses to nivolumab. This study aims to evaluate the efficacy and safety of nivolumab monotherapy by performing retrospective analyses of patient data. The data was collected from HCC patients attending three university-affiliated hospitals in Korea where HBV infection is endemic. Specifically, we focused on the responses to nivolumab monotherapy, for every tumor in a single patient, to identify the factors associated with the heterogeneous responses to this treatment.

## Methods

### Study Design and Population

The Institutional Review Board of The Catholic University of Korea approved this study (Xc20RIDI0015), which was carried out in accordance with the Declaration of Helsinki. Data was collected between October 2016 and November 2019 from 33 consecutive patients treated at three university-affiliated hospitals in Korea. Among the enrolled patients, 31 patients were enrolled between February 2018 and November 2019. All patients had a verified diagnosis of unresectable HCC by updated international guidelines (17, 18) and were treated with nivolumab. Experienced hepatologists reviewed the patients' medical data. The survival data of the patients continued to be followed-up until February 2020. Survival was determined to be from the point of commencing nivolumab treatment until the final follow-up or until the patient died, regardless of the cause. The inclusion criteria were a diagnosis of the inoperable HCC treated with nivolumab. Albumin–Bilirubin (ALBI) grade (19) was calculated to determine the liver reserve of patients treated with nivolumab.

### Nivolumab Treatment and Response Evaluation

Each patient received an intravenously delivered dose of 3 mg/kg nivolumab (OPDIVO®, Bristol-Myers Squibb) every two weeks. Every 4 to 8 weeks during treatment, full blood counts were performed, and a number of markers were evaluated including alpha-fetoprotein (AFP), alanine aminotransferase (ALT), bilirubin, and prothrombin time. Nivolumab was administered according to the recommended dose and safety information. Where necessary, doctors would adjust the treatment schedules. Toxicities of nivolumab were diagnosed and managed as previously described (20, 21).

Using the modified Response Evaluation Criteria in Solid Tumors (mRECIST) tool, two independent radiologists assessed the response to the treatment, as described elsewhere (3). A maximum of two lesions per organ and five in total were chosen for the evaluation of the treatment responses by mRECIST (3, 22). Extrahepatic tumors exhibiting enhanced contrast were considered as target lesions, whereas macroscopic vascular invasions were regarded to be non-target lesions. The mRECIST tool categorizes a complete response (CR) when the intratumoural arterial enhancement disappears from all tumors. A partial response (PR) is defined when the sum of the diameters of enhanced lesions are reduced by no <30%. However, if the sum of the diameters of enhancing lesions increases by 20% or more, the disease is categorized as progressive (PD). Disease states not categorized part of PD and PR, were determined to be stable (SD) (3, 22). The sum of CR, PR and SD rates formed the disease control rate (DCR). The response evaluation was conducted regularly, between two and four nivolumab-treatment cycles.

### Statistical Analysis

For the statistical analyses, SPSS version 26 software (IBM Corp., Armonk, NY, USA) was used. A chi-square test was used to analyse the two groups' categorical variables, and an independent *t*-test was conducted to evaluate the continuous variables. Univariate and multivariate analyses were performed to establish prognostic factors of OS. For the univariate analyses, such as survival probabilities, the Kaplan-Meier method and log-rank tests were used. Factors that were significant in the univariate analysis at *P* < 0.05 were advanced to the multivariate analysis, which was undertaken using a Cox regression model.

## Results

### Study Cohort Demographics

As indicated in Table 1, the study involved a total of 33 patients, 25 of whom were male (76%). Ages ranged from 37-79 years, with a median of 57 years. A majority of patients (88%) had been assigned stage C on the Barcelona Clinic Liver Cancer (BCLC) staging system, with a median tumor size of 3.5 cm. Extrahepatic metastases were reported in 26 patients (79%), and portal vein tumor thrombosis were detected in 10 patients (30%). The most prevalent underlying liver diseases was chronic HBV infection, which affected 29 individuals (88%). A majority of participants (79%) were classified as Child–Pugh class A at the time of enrolment, and 15 patients (45%) were ALBI grade 1. The median level of AFP was 665 ng/mL (normal range: <8.1 ng/mL), and the level of 17 patients (52%) were above 1000 ng/mL. Most of the enrolled patients (94%) underwent sorafenib treatment, and 13 patients (39%) patients underwent regorafenib treatment prior to the nivolumab therapy. Prior to the systemic therapy, most patients had undergone local–regional therapies such as trans-arterial chemoembolization or hepatic arterial infusion chemotherapy. Eleven patients received further treatments after nivolumab, which included cabozantinib and regorafenib. In this cohort, there was no evidence of a high incidence of immunotherapy-related adverse events. Grade 3 toxicities occurred in one patient, who developed pneumonitis after 5 cycles of nivolumab treatment.

**Table 1:** Clinical parameters of study patients.

**Clinical parameters**	***n* = 33**
Median age (range)	57 (37–79)
Sex (male), *n* (%)	25 (75.8)
HBsAg-positivity, *n* (%)	29 (87.9)
Anti-HCV-positivity, *n* (%)	1 (3)
Median tumor size, cm	3.5
<5 cm, *n* (%)	21 (64)
≥5 cm, *n* (%)	12 (36)
Multiple tumors, *n* (%)	33 (100)
Portal vein tumor thrombosis, *n* (%)	10 (30)
Extrahepatic metastasis, *n* (%)	26 (79)
BCLC stage B/C, *n* (%)	4/29 (12/88)
Median AFP (range), ng/mL	665 (1.3–160000)
<1000 ng/mL, *n* (%)	17 (52)
≥1000 ng/mL, *n* (%)	16 (49)
Child–Pugh score	
5, *n* (%)	20 (61)
6, *n* (%)	6 (18)
7, *n* (%)	7 (21)
ALBI grade 1/2/3, *n* (%)	15/18/0 (45/55/0)
Prior therapy to nivolumab, *n* (%)	
Surgical resection	12 (36)
TACE / TARE	26 (79)
HAIC	5 (15)
Sorafenib	31 (94)
Regorafenib	13 (39)
Lenvatinib	2 (6)
Post nivolumab treatment, *n* (%)	
No treatment	21 (64)
Resection	1 (3)
TACE	2 (6)
Radiation therapy	3 (9)
Regorafenib	2 (6)
Cabozantinib	2 (6)
HAIC	1 (3)
Systemic chemotherapy	2 (6)
Best responses to nivolumab	
Complete response	2 (6)
Partial response	4 (12)
Stable disease	4 (12)
Progressive disease	19 (58)
Not assessed	4 (12)

*AFP, alpha fetoprotein; ALBI grade, albumin-bilirubin grade; BCLC stage, Barcelona-Clinic liver cancer stage; HAIC, hepatic arterial infusion chemotherapy; HBsAg, hepatitis B surface antigen; HCV, hepatitis C; RT, radiotherapy; TACE, transarterial chemoembolization; TARE, transarterial radioembolization*.

### Treatment Responses to Nivolumab

**Figure 1:**
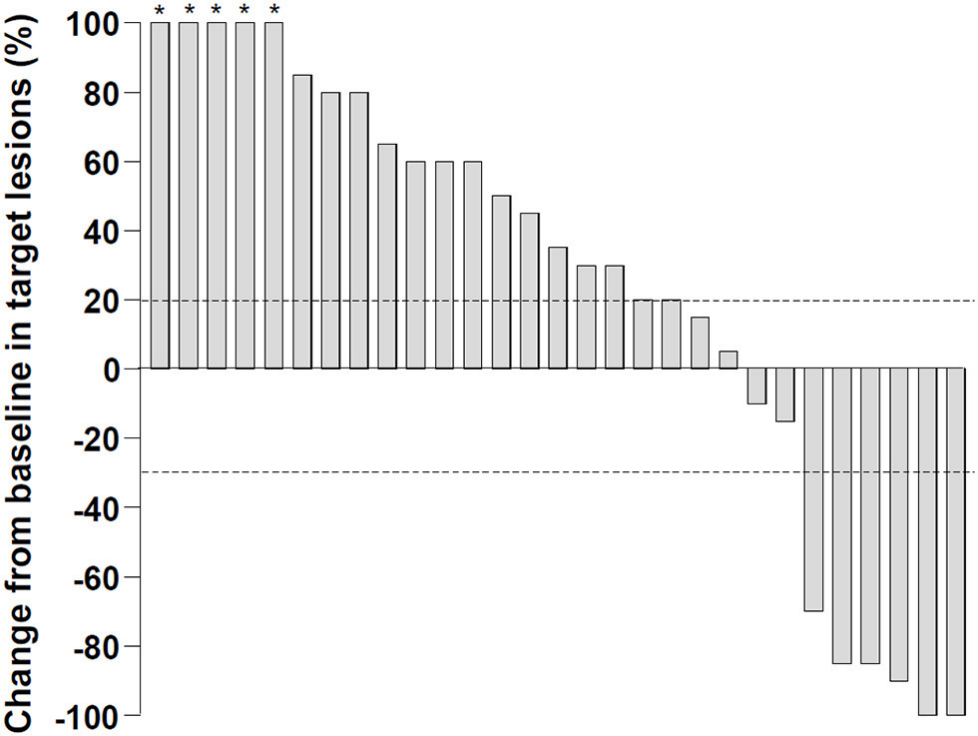
Changes in target lesions from the baseline after nivolumab monotherapy. Dashed lines represent a 20% increase or a 30% reduction. The percentage changes more than 100% were truncated to 100% (asterisks) .

In this study, the nivolumab monotherapy was administered for 2 to 160 weeks, with a median of 8 weeks, and the number of treatment cycles varied from 1 to 78, with a median to 3 cycles. In a best response evaluation after nivolumab administration, 2 patients exhibited a CR according to the mRECIST (Table 1). Four patients displayed a PR and 4 patients displayed a SD. However, 4 patients did not undergo imaging for the response evaluation. The objective response rate was 18% among all the patients that enrolled in this study, and 24% among patients with evaluable images. The median duration of treatment responses by nivolumab was 13.3 months. The disease control rate was 30% in our cohort. A waterfall plot describes the marked reductions in target lesions from baseline tumor burden in patients with objective responses to nivolumab monotherapy (Figure 1).

### Factors Associated With the Overall Survival

**Figure 2:**
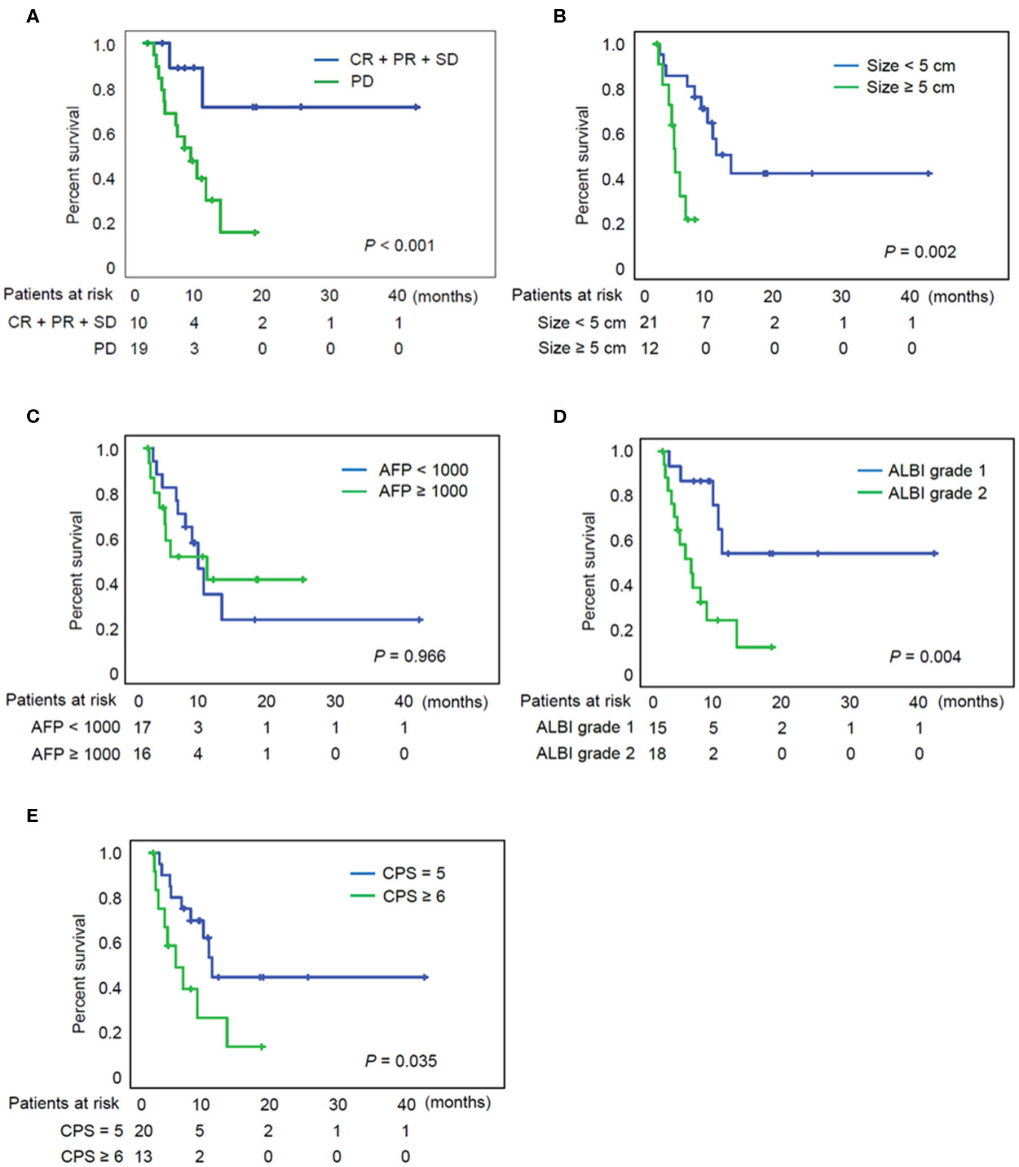
Overall survival of patients according to the various clinical parameters. **(A)** Overall survival of patients according to the tumor response. **(B)** Overall survival of patients according to the tumor size. **(C)** Overall survival of patients according to the AFP. **(D)** Overall survival of patients according to the ALBI grade. **(E)** Overall survival of patients according to the CPS. AFP, alpha fetoprotein; ALBI grade, albumin-bilirubin grade; CPS, Child-Pugh score; CR, complete response; PD, progressive disease; PR, partial response; SD, stable disease.

The median follow-up period after initiation of treatment was 12.5 months, and the OS ranged from 2.3 to 175.1 weeks (median: 26.4 weeks). At the time when data analysis was performed (February 2020), 18 of the 33 patients (55%) had died from causes such as tumor progression, variceal bleeding, or fatal systemic infection. Figure 2A indicates a significantly better OS for individuals with controlled diseases (CR + PR + SD) than for those who displayed PD (log rank test, *P* < 0.001). Figure 2B indicates a significantly better OS for individuals with a maximal tumor size of < 5 cm (*P* = 0.002), although AFP level did not have a significant impact on the patient survival (Figure 2C). Figure 2D indicates a better OS for individuals with ALBI grade 1 than for those with grade 2 with *P* = 0.004. Patients with Child–Pugh score 5 also showed superior OS to those with score 6 (Figure 2E, *P* = 0.035).

The prognostic factors for OS after nivolumab treatment are presented in Table 2. These parameters were subjected to univariate analyses initially, and tumor size / ALBI grade were included in a subsequent multivariate Cox regression model. The favorable prognostic factors for OS were a tumor size <5 cm, the assignment of ALBI grade 1, and Child-Pugh score 5 in univariate analyses. In multivariate analysis, tumor size <5 cm (HR = 0.269; *P* = 0.034) and ALBI grade 1 (HR = 0.312; *P* = 0.04) were both significant factors for OS.

**Table 2:** Factors associated with overall survival in 33 patients treated with nivolumab monotherapy.

**Characteristics**		**Univariate**			**Multivariate**		
		**HR**	**95% CI**	***P***	**HR**	**95% CI**	***P***
Age	<60 vs. ≥60	2.065	0.734–5.807	0.169			
Sex	Male vs. Female	0.457	0.151–1.384	0.166			
**Tumor size, cm**	**<5 vs**. **≥5**	**0.181**	**0.056–0.586**	**0.004**	**0.269**	**0.080-0.906**	**0.034**
PVTT	Yes vs. No	1.868	0.695–5.020	0.215			
AFP, ng/mL	<1000 vs. ≥1000	0.980	0.382–2.511	0.966			
Child–Pugh score	5 vs. 6	0.380	0.150–0.966	0.042			
**ALBI grade**	**Grade 1 vs. 2**	**0.236**	**0.083–0.675**	**0.007**	**0.312**	**0.103-0.949**	**0.040**

*AFP, alpha fetoprotein; AFP, alpha fetoprotein; ALBI grade, albumin-bilirubin grade; CI, confidence interval; HR, hazard ratio. Significant factors in multivariate analysis are in bold characters*.

### Differential Responses to Nivolumab Among Multiple Tumors in Each Patient

**Figure 3:**
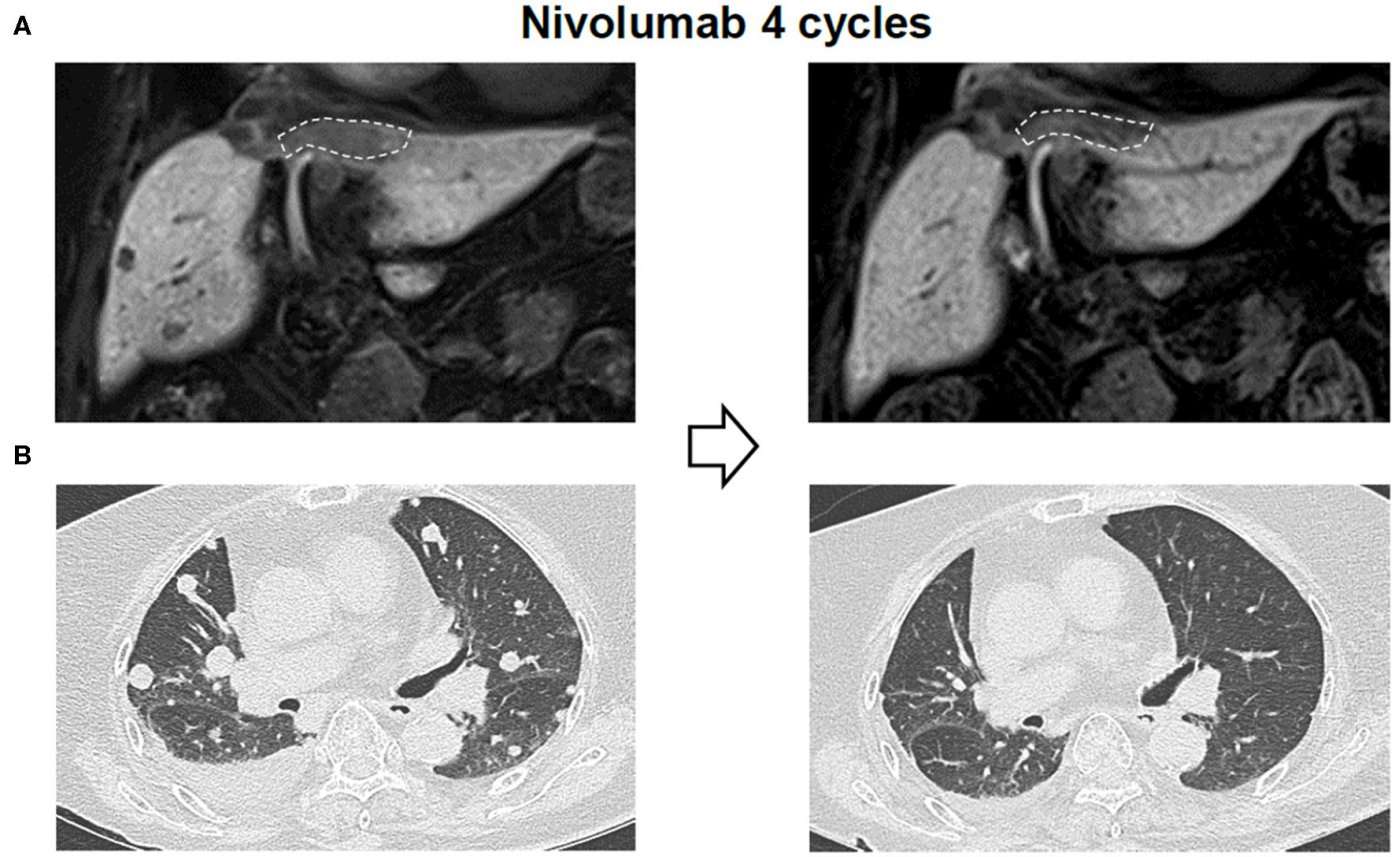
Representative imaging data of a patient with heterogeneous responses to nivolumab. **(A)** Liver imaging findings after 4 cycles of nivolumab. **(B)** Lung imaging findings after 4 cycles of nivolumab.

Due to the previous demonstration that maximal tumor size is a critical factor of OS of patients with nivolumab treatment, we measured response of each tumor among multiple tumors in a single patient. Heterogeneous responses were detected in 6 patients among the 33 enrolled patients (18%) (Table 3). For patient #2, the different tumor responses between lung (1.2 cm) and peritoneal metastasis (4.3 cm) and the heterogeneous responses to nivolumab treatment was also noted even within the single peritoneal metastatic nodule. This case was previously reported by our group (16). In the peritoneal metastatic nodule, metastatic HCC with partial necrosis was present with viable tumor cells with various types of tumor-infiltrating immune cells, suggesting the immune heterogeneity within a single tumor when it exhibits a considerable size. Figure 3 shows the imaging findings of patient #6 after 4 cycles of nivolumab. Intrahepatic infiltrative tumor (Figure 3A) showed slight increase in its extent in hepatobiliary phase of primovist-enhanced magnetic resonance imaging, while metastatic nodules in lung showed dramatic responses after nivolumab (Figure 3B).

**Table 3:** Patients with heterogeneous responses to nivolumab.

	**Intrahepatic tumor**		**Extrahepatic tumor−1**			**Extrahepatic tumor−2**			**Overall response**
**Pt**.	**Size (cm)**	**Response**	**Location**	**Size (cm)**	**Response**	**Location**	**Size (cm)**	**Response**	
#1	No tumor		Lung	4.2	10% ↑	Lung	2.6	60% ↓	PR
#2	No tumor		Peritoneum	4.3	13% ↑	Lung	1.2	80% ↓	PR
#3	1.4	5%↓	Lung	1.8	10% ↑	Lung	1.2	30% ↓	SD
#4	3.3	100% ↑	Lung	1.2	70% ↓	Lung	1	80% ↓	SD
#5	3.3	25%↓	Peritoneum	1.0	200%↑	Lung	1	100%↑	PD
#6	7.2	5% ↑	Lung	2.1	55% ↓	Lung	1.9	62% ↓	PR

*PD, progressive disease; PR, partial response; SD, stable disease*.

## Discussion

This is the first report in Korea demonstrating the potential predictors of OS in patients treated with nivolumab for unresectable HCC. In this study, we investigated the safety, efficacy, and the potential predictors of OS in nivolumab monotherapy for unresectable HCC in an endemic area of HBV infection. Moreover, we demonstrated the striking heterogeneous responses to nivolumab monotherapy in a single patient with multiple tumors according to each tumor size. The median OS of the participating patients (26.4 weeks) was longer than the recently conducted real-world study in Europe (34 enrolled patients, OS: 7.5 weeks), which included many patients with poor liver function (41% with Child Pugh class B) (23), but shorter than the Taiwanese real-world study (92 patients with nivolumab treatment and 3 patients with pembrolizumab treatment, OS: 11.9 months) (24). As expected, it was also shorter than that of the ITT analysis comprising an Asian cohort of CheckMate-040 (14.9 months) (14). As nivolumab is approved only in patients with previous sorafenib failure in Korea and not reimbursed by the government insurance system, Korean HCC patients receive nivolumab treatment as the last possible option for the advanced HCC. Furthermore, shorter follow-up duration may also have affected the shorter OS of our real-world data than those of previous clinical trial data.

In our study, maximal tumor diameter was the significant pre-treatment factor that affected the OS in multivariate analyses. Previous report demonstrated that the ratio of T-cell invigoration to tumor burden ratio correlates with the response to pembrolizumab in melanoma patients (25, 26). Other clinical studies showed that tumor size is an independent factor for OS in melanoma and non-small cell lung cancer patients treated with nivolumab or pembrolizumab (27–29). In HCC, very recent data using multi-omics approaches demonstrated the significant heterogeneity of tumor cells in HCC, while the heterogeneity of immune microenvironment was not as dramatic (15). In line with these reports, differential responses to nivolumab among multiple tumors in a patient with HCC can be understood. Heterogeneous responses to nivolumab among multiple metastatic tumors were also demonstrated in melanoma (30) and non-small cell lung cancer patients (31). This heterogeneity can be explained by innate or acquired resistance of tumor cells when a considerable tumor burden exists. In this report, we observed that each tumor size in a patient with multiple tumors may be associated with the heterogeneous responses to nivolumab. In HCC, immune heterogeneity may be applied to the larger tumors that may contain the higher number of resistant clones to immune checkpoint inhibitors. To overcome this heterogeneity, there have been studies investigating the possible synergic benefits for advance HCC of combination therapy (32). Lenvatinib plus pembrolizumab or bevacizumab plus atezolizumab has demonstrated promising objective response rates (ORRs). A study at the European Society for Medical Oncology Asia Congress 2019 showed significant improvements of atezolizumab and bevacizumab over sorafenib in OS and recurrence-free survival for unresectable HCC (phase 3 IMbrave 150) (32). This suggests that resistance to immune checkpoint inhibitors can be overcome by the combination with anti-angiogenic drugs or tyrosine-kinase inhibitors.

Patients in the real-world cohorts are typically more heterogeneous than those recruited into clinical trials. It is currently still unclear whether nivolumab offers any OS benefit to patients with decreased liver function. There was also a limited treatment effect to these patients in this study. The data from our cohort confirmed the ALBI grade as an independent survival predictor in patients undergoing nivolumab treatment. The results of our survival analysis indicate that ALBI grade 1 is an independent factor for the favorable survival. This is in accord with the previous study demonstrating the survival-predictable ability of the ALBI grade at the time of sorafenib discontinuation (33).

This study has a number of limitations. This was a small-sized retrospective study that used patients only from three facilities. Such a small number of the cohort is not sufficient to validate the safety and efficacy of the drug. Moreover, regular tumor reassessment by clinical and imaging evaluation would have decreased the observation bias. Lastly, since liver biopsy was not performed routinely before nivolumab treatment, the molecular biomarkers for nivolumab responses were not studied.

In conclusion, our study demonstrates that nivolumab monotherapy is clinically efficacious in treating unresectable HCC in an endemic area of HBV infection. Maximal tumor diameter and the indicator of liver function (ALBI grade) were significant factors in multivariate analyses that predicted the OS of HCC patients treated with nivolumab monotherapy. Future prospective study is required to overcome the probable heterogeneous efficacy of nivolumab monotherapy according to each tumor size.

## Data Availability Statement

The original contributions presented in the study are included in the article/supplementary materials, further inquiries can be directed to the corresponding author/s.

## Ethics Statement

The studies involving human participants were reviewed and approved by the Institutional Review Board of the Catholic University of Korea (Xc20RIDI0015). The patients/participants provided their written informed consent to participate in this study.

## Author Contributions

PS: study design, data collection, data analysis, data interpretation, article, and article approval. JL, SKL, HL, HY, HN, and SWL: data collection. JJ, SB, JC, NH, and SY: data interpretation and article approval. All authors contributed to the article and approved the submitted version.

## Conflict of Interest

The authors declare that the research was conducted in the absence of any commercial or financial relationships that could be construed as a potential conflict of interest.
